# Semantic-enhanced graph neural network for named entity recognition in ancient Chinese books

**DOI:** 10.1038/s41598-024-68561-x

**Published:** 2024-07-30

**Authors:** Yongrui Xu, Caixia Mao, Zhiyong Wang, Guonian Jin, liangji Zhong, Tao Qian

**Affiliations:** 1 School of Computer Science and Technology, Hubei University of Science and Technology, Xianning, 437100 China; 2 School of Electronics and Information Engineering, Hubei University of Science and Technology, Xianning, 437100 China

**Keywords:** Named entity recognition, Graph neural network, Ancient Chinese, Graph attention mechanism, Computer science, Information technology

## Abstract

Named entity recognition (NER) plays a crucial role in the extraction and utilization of knowledge of ancient Chinese books. However, the challenges of ancient Chinese NER not only originate from linguistic features such as the use of single characters and short sentences but are also exacerbated by the scarcity of training data. These factors together limit the capability of deep learning models, like BERT-CRF, in capturing the semantic representation of ancient Chinese characters. In this paper, we explore the semantic enhancement of NER in ancient Chinese books through the utilization of external knowledge. We propose a novel model based on Graph Neural Networks that integrates two different forms of external knowledge: dictionary-level and chapter-level information. Through the Graph Attention Mechanism (GAT), these external knowledge are effectively incorporated into the model’s input context. Our model is evaluated on the C_CLUE dataset, showing an improvement of 3.82% over the baseline BAC-CRF model. It also achieves the best score compared to several state-of-the-art dictionary-augmented models.

## Introduction

Ancient Chinese books, such as “Dream of the Red Chamber” and “Twenty-Four Histories”, embody a wealth of historical, cultural, and societal information, and are a vital source of information for studying and understanding Chinese history and culture. However, due to the evolution of language and script, the textual content in ancient Chinese books has become challenging to comprehend and apply, limiting their utilization and dissemination in modern society and research^1,2^.

Named Entity Recognition (NER), as a fundamental task in Natural Language Processing (NLP), holds significant importance for information extraction in ancient Chinese books^3,4^. Through NER, we can accurately identify and extract specific categories of information, such as personal names, location names, and organization names, which plays a crucial role in the extraction and utilization of knowledge of ancient Chinese books.

Chinese NER is a very challenging task due to the lack of word separators in Chinese. Compared with NER in modern Chinese, NER in ancient Chinese is more difficult. One of the reasons is the concise nature of ancient texts, often characterized by single characters and short sentences, which frequently results in semantic ambiguity. This ambiguity makes it challenging to capture the semantic of these texts with current technologies, such as neural networks or deep learning models, thus complicating the NER process. Secondly, the scarcity of training data for ancient texts limits the ability of deep learning models to fully understand the complex meanings of ancient Chinese characters. Although pre-trained language models have significantly improved the semantic representation of texts, the challenges of performing NER in ancient texts with limited resources persist.

An effective method to enhance semantic representation is to utilize external knowledge, such as Part of Speech^5^, syntactic information^6^ and dictionary^7,8^, which have been proven to be useful in NER task. In particular, dictionary contains rich lexical information and can improve semantic representation when training resources are limited. Zhang et al.^7^ encoded all potential words matching a modern Chinese sentence into the lattice-structured Long Short-term Memory Networks (LSTM). Wu et al.^8^ integrated the all “n-gram” words via into deep neural networks for Chinese clinical named entity recognition. These methods achieved improved results on various datasets due to its rich lexical information.

**Figure 1:**
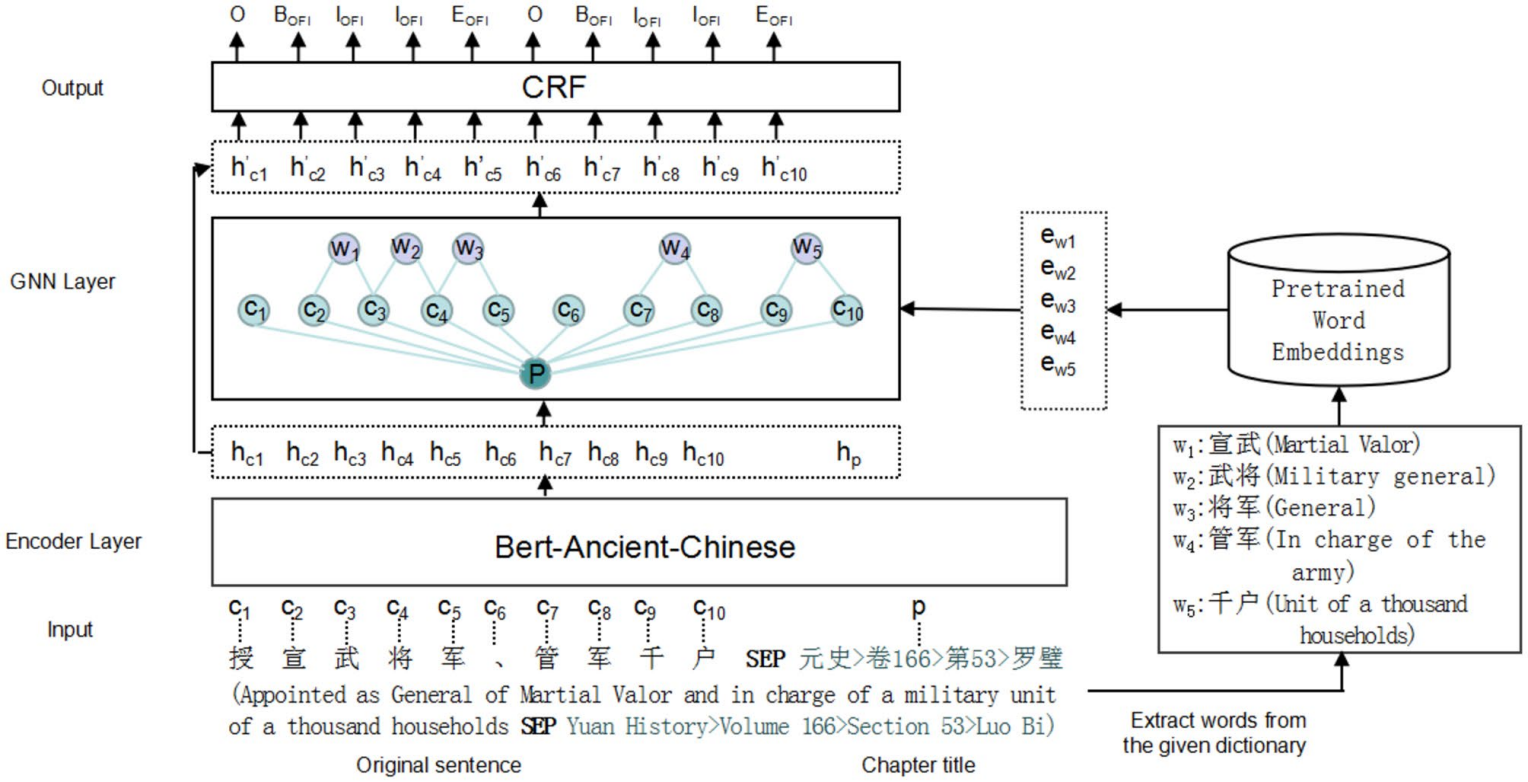
The figure illustrates the architecture of our BAC-GNN-CRF NER model with an example. The input consists of an ancient Chinese sentence ($\ldots , c_i,\ldots$) and its corresponding chapter title (*P*) concatenated with *SEP*. The encoder layer generates contextualized embeddings ($h_{ci}, h_p$) for each character and the chapter title using the “Bert-Ancient-Chinese” model. In the GNN layer, the vertex set of the graph consists of the Chinese characters ($c_i$), matching words ($w_i$), and the chapter (*P*, serving as the global node). The words are extracted from a dictionary. The global node links all character nodes, while the word nodes link their corresponding character nodes. The CRF layer produces the final output tags for each character in the input sentence. Each tag consists of a prefix and its entity type (e.g. $B_{OFI}$ denotes the beginning of an Office entity). The prefixes B-I-E-O stand for Begin, Inside, End, and Outside, respectively. The right part shows the process of incorporating matching words.

In this paper,we explore the utilization of external knowledge to enhance NER in ancient Chinese books. We propose a novel model based on Graph Neural Networks (GNN) specifically designed for this task. The structure of our model is illustrated in Fig. 1. The encoder layer employs the pre-trained language model Bert trained on ancient Chinese texts^9^ (Bert-Ancient-Chinese, BAC) and the output layer uses the standard condition random field (CRF). Our approach enriches the model by embedding a GNN layer between the encoder and output layers, facilitating the incorporation of external knowledge. This is accomplished by constructing a character–word graph that injects rich lexical information corresponding to the input sentences. Moreover, acknowledging the significant role of chapter titles in ancient books - which often encapsulate the essence of the chapters and aid in resolving semantic ambiguities - our model integrates the information from chapter titles into its graph structure. This integration involves including the chapter title text associated with the input sentence as part of the model’s input, subsequently encoding it, and then treating it as a global node within the graph structure. To further enhance the model’s contextual understanding with external knowledge, we employee a Graph Attention Mechanism (GAT), ensuring an effective assimilation of these knowledge into the main information.

Our experiments conducted on the available dataset for entities in ancient books demonstrate that incorporating both dictionary and chapter title information significantly enhances the performance of our model. The key contributions of our research are outlined as follows:

1. We propose a GNN-based model, namely BAC-GNN-CRF, specifically designed for NER in ancient Chinese books by integrating external knowledge.
2. We utilize graphs to incorporate two types of external knowledge - dictionary and chapter information - with the goal of improving semantic representation and diminishing ambiguity.
3. Experimental results demonstrate our model is highly effective, showcasing consistent improvements and leading to state-of-the-arts in the field on the evaluated dataset.

## Related work

Early named entity recognition (NER) primarily depended on rule-based methods and statistical machine learning approaches^10^. However, the emergence of neural networks marked a significant advancement in this field. Models such as BiLSTM-CRF and BERT-CRF have notably enhanced NER capabilities, demonstrating improved performance across diverse benchmark evaluations^11,12^.

### Low-resource NER

The huge training data required by neural networks has led to a decline in performance for Low-resource NER, such as biomedical, social media, etc. A number of recent technologies have been proposed to tackle the low-resource domain NER, such as pre-trained embedding^13^, multi-task^14^, multi-models^15^, transfer learning^16^.

On the other hand, Numerous studies have shown that incorporating additional useful knowledge, such as external dictionaries, can still achieve significant gains in NER tasks. Nie et al.^17^ proposed a semantic expansion module that encodes dictionary information, assigning different weights to each word matched in the dictionary and effectively alleviating the data sparsity issue in social media texts. Diao et al.^18^ enhanced the pretrained language model BERT by externally adding an encoder to handle N-Gram dictionary, thereby explicitly integrating lexical-level information. Liu et al.^19^ integrated dictionary information into the underlying part of BERT, enabling BERT to learn deeper knowledge from the dictionary. This integration resulted in excellent performance in NER and various sequence labeling tasks. Nevertheless, adding dictionary information to the base layer of pre-trained language models raises their complexity, posing practical application challenges. In contrast, our approach avoids embedding knowledge directly into the core of the pre-trained language model. We opt for adding a graph neural network layer, which streamlines the training process of the NER model.

### Graph neural networks on NER

Numerous recent methods leveraging Graph Neural Networks (GNNs) have emerged for Named Entity Recognition (NER)^20–22^. Chen et al.^20^ presented a Randomly Wired Graph Neural Network specifically designed for Chinese NER, pioneering a novel context encoder that automatically generates the wiring pattern of the graph network. Sui et al.^21^ introduced collaborative Graph Attention Networks^23^ to integrate self-matched lexical knowledge in Chinese NER, aiming to resolve ambiguities inherent in Chinese text. Wang et al.^22^ proposed a Polymorphic Graph Attention Network, which uses softLexicon^24^ to construct graphs between characters and matched words and capture correlation from them. Our work is closely related to the graph attention networks to ensure the efficient integration of these knowledge into the model’s input context.

### NER in ancient Chinese

Existing research on NER in ancient Chinese has progressively shifted towards leveraging deep neural network models. Xu et al.^25^ utilized basic neural network models such as BiLSTM-CRF and BERT to explore the effects of entity recognition within the “Fang Zhi Wu Chan” Yunnan volume corpus. Liu et al.^26^ proposed a semi-supervised learning method combined with feature words for medicine NER in traditional Chinese medicine ancient text. Zhang et al.^27^ explored NER in ancient Chinese wine texts using deep learning models, particularly focusing on BERT pre-training models.

To enhance the representation of ancient texts, researchers have proposed several pre-trained language models based on ancient text corpora, such as SikuBERT^28^, GuwenBERT (GuwenBERT https://github.com/ethan-yt/guwenbert), Bert-Ancient-Chinese (BAC)^9^. Feng et al.^29^ proposed a classical Chinese named entity recognition model, which is based on Bert-ancient-Chinese+Recurrent Long Short-Term Memory+Conditional Random Field (BAC+RLSTM+CRF) for Named Entity Recognition and tested on the C-CLUE dataset. Ge Sijia^30^ adopt the SikuBERT model as the pre-trained model to integrate both named entity recognition and sentence segmentation tasks. In this paper, we utilize Bert-Ancient-Chinese as the encoder and integrate external dictionary and chapter information to further enhance the representation of ancient texts.

## Method

Figure 1 illustrates the main architecture of our model, which is partitioned into four layers: the input, the encoder, the GNN and the output. In this section, we meticulously detail each component that comprises our model, subsequently unfolding the methods involved in the training and inference phases of the model.

### Input

For the NER task, the input is a given ancient sentence $S=c_1, c_2, \ldots , c_n$, where $c_i$ is the $i_{th}$ character and *n* is the length of the input sentence, and the output is the predicted entity labels $Y=y_i,\ldots , y_n$. In our model, besides the sentence *S* itself, we also introduce two types of knowledge as inputs: the lexical sets $L_s$ matching the input *S* and the global chapter information *P*.

#### Acquisition of lexical sets

Integrating rich lexical information into models is a promising approach for enhancing named entity recognition in ancient Chinese. Given a input sentence *S* and the dictionary knowledge base, we use a full matching method to identify all potential lexical items within the sentence over the dictionary, formulated as:

1 $$\begin{aligned} (w_1, w_2,\ldots , w_m) = Matching(S). \end{aligned}$$

where $w_i$ is the matching lexical item. An example of this is demonstrated in Fig. 1, we extract five lexical items. The semantic information of these lexical items will be incorporated into a graph neural network, thereby infusing the model with a deeper understanding of lexical knowledge.

#### Acquisition of chapter information

Ancient texts frequently feature short sentences and single-character words, which significantly increases the ambiguity in entities. It’s commonly observed that an entity, when mentioned multiple times throughout a chapter, retains the same meaning. Chapter titles in ancient books refer to the themes and structures of the chapter text, hence integrating chapter information into sentences should help to eliminate ambiguity of entity. To achieve this, we introduces a technique where chapter information are appended directly to the input sentences.

To link input sentences within the same chapter, this paper seeks the origins of each sentence in the Chinese Ancient Books Library (https://publish.ancientbooks.cn/docShuju/platform.jspx). In Fig. 1, take the sentence *S* “授宣武将军、管军千户 (Appointed as General of Martial Valor and in charge of a military unit of a thousand households)” as an example: by querying the website, we identify that it belongs to the chapter *P*: “元史>卷166>第53>罗璧 (Yuan History>Volume 166>Section 53>Luo Bi)”. We then add this chapter information as an extension to the input sentence:$S\#P$. Once encoded, the chapter embedding $h_p$ serves as a global node within the graph neural network, incorporating comprehensive global context.

### Encoding

The model’s input is composed of three elements: the input sentence *S* for prediction, the associated prior chapter information *P*, and the matching lexical set $L_s$. As shown in Fig. 1, we concatenate the input sentence *S* with the prior chapter information *P*. Then the concatenated sequence $S \# P$ is fed into a pre-training language model **(Bert-Ancient-Chinese, BAC)** for encoding, treating *P* as the next sentence to *S*. Through the encoding process, the pre-training language model is able to incorporate the chapter information into the input sentence. The coding is formulated as follows:

2 $$\begin{aligned} h_1, h_2,\ldots , h_p = BAC(c_1, c_2,\ldots , c_n\#P) \end{aligned}$$

Additionally, to incorporate an abundance of external information, the matching dictionary is encoded using embeddings trained externally, the encoding is as follows:

3 $$\begin{aligned} e_1, e_2,\ldots , e_m = Embeddings(w_1, w_2,\ldots , w_m) \end{aligned}$$

where *m* is the number of words matching lexical sets. In the experiment, we utilize vocabulary embeddings developed by Tencent (https://ai.tencent.com/ailab/nlp/en/download.html). Although these embeddings are primarily trained using modern texts, their semantics have evolved from classical literature. Integrating this evolved semantic information should also enrich the semantic representation of ancient texts, which has been validated in the experimental section.

### Graph neural network

#### Graph construction

We need to construct a graph for integrating both the matching lexical items and chapter information into the input sentence. The vertex set of a graph is made up of the Chinese characters in the input sentence, lexical items extracted from it, and the chapter. For example, as shown in Fig. 1, the vertex set is $V=V_{char}\{c_1, c_2, \ldots , c_{10}\} \cup V_{lex}\{w_1, w_2, \ldots , w_6\} \cup V_{chapter}\{P\}$.

To represent the edge set, the adjacency matrix needs to be introduced. The elements of the adjacency matrix indicate whether pairs of vertices are adjacent or not in the graph. if a lexical item *i* contains a Chinese character *j*, the *edge*(*i*, *j*) will be assigned a value of 1. Intuitively, character-lexical item pairs can capture the semantic and boundaries information of words in the sentence, which is help for named entity recognition. For vertex chapter *P*, it is connected to each character vertex, which is regarded as a global node and can more effectively infuse chapter information.

#### Graph attention

The matching lexical set may include some noise words, which mainly refer to words that are incorrectly segmented by the full-match word segmentation method. Such words do not contribute to the main information and may even have a negative impact. For example, in Fig. 1, the lexical item “武将(military general)” is a noise, and its integration will affect the semantic expression of the sentence. In order to alleviate the impact of noise words, we employ the Graph Attention Network (GAT) for modeling on the graph. The initial input to the graph is a set of node features from encoding layer $NF^0 = {h_1, h_2,\ldots , h_n, h_p, e_1, e_2,\ldots , e_m }$ where *n* is the node number of input sentence, *m* is the node number of the matching lexical items and the total nodes is $N=m+n+1$. In an M-layer GAT, the input of *l*-th layer is a set of node features, $NF^l = {h_1^l, h_2^l,\ldots , h_{N}^l }$, together with an adjacency matrix *A*, $h_i \in R^{F^l}$ , $A \in R^{N\times N}$, where $F^l$ is the dimension of features at *l*-th layer. The output of *l*-th layer is a new set of node features, $NF^{l+1}=\{h_1^{l+1}, h_2^{l+1},\ldots , h_N^{l+1}\}$, which are regarded as the input of $(l+1)$-th layer. We employ multi-head attention to compute the node features. Specifically, a *l*-th layer GAT operation with *K* independent attention heads can be formulated as :

4 $$\begin{aligned} h_i^{l+1}=\oplus _{k=1}^{K}\sigma \left( \sum _{j\in \mathbb {N}_i} \alpha _{ij}^k W_k h_j^l\right) \end{aligned}$$

5 $$\begin{aligned} \alpha _{ij}^k=\frac{exp(LeakyReLU(a^T[W^kh_i^l]\oplus W^kh_j^l))}{\sum _{k\in \mathbb {N}_i}exp(LeakyReLU(a^T[W^kh_i^l]\oplus W^kh_j^l))} \end{aligned}$$

where $\oplus$ denotes concatenation operation, $\sigma$ is the ReLU (Rectified Linear Unit) non-linear activation function, $\mathbb {N}_i$ is the neighborhood of node *i* in the graph, $\alpha _{ij}^k$ are the attention coefficients, $W^k \in R^{F^{l+1}\times F^l}$, and $a \in R^{2F^{'}}$ is a single-layer feed-forward neural network. Note that, the dimension of the output $h_i^{j+1}$ is $K*F^{l+1}$. At the last layer, the dimension of final output features is $h^{'}$, which are computed using averaging operation.

6 $$\begin{aligned} h_i^{'}=\sigma \left( \frac{1}{K}\sum _{K}^{k=1}\sum _{j\in \mathbb {N}_i} \alpha _{ij}^kW^kh_j^M\right) \end{aligned}$$

where $\sigma$ is also the ReLU function

The output of all node features is denoted as *G*,

7 $$\begin{aligned} G=GAT(V, A) \end{aligned}$$

where $G \in R^{F^{m}\times (n+m+1)}$. We keep the first n columns of these matrices and discard the last $m+1$ columns, because only character representations are used to decode labels.

8 $$\begin{aligned} Q=G[:, 0:n] \end{aligned}$$

Finally, the input of CRF layer is denoted as:

9 $$\begin{aligned} R= W_1H + W_2Q \end{aligned}$$

where $W_1$ and $W_2$ are trainable matrices. The new represent R for sentence integrate the contextual information from encoding layer and the semantic information of the lexical set and chapter from GNN layer.

### Decoding and training

A standard CRF layer is used to capture the dependencies between successive labels. Formally, we took the above $R = \{r_1, r_2, \ldots , r_n\}$ as our input to the CRF layer, and its output was the conditional probability of the golden tag sequence $y = \{l_1, l_2, \ldots , l_n\}$ is

10 $$\begin{aligned} p(y|s) =\frac{exp(\sum _i(W_{CRF}^{l_i}r_i+T_{CRF}^{l_{i-1},l_i}))}{\sum _{y^{'}}exp(\sum _i(W_{CRF}^{l_i^{'}}r_i+T_{CRF}^{l_{i-1}^{'},l_i^{'}}))} \end{aligned}$$

here $y^{'}$ is an candidate label sequence, $W_{CRF}^{l_i}$ is used for modeling emission potential for the *i*-th word in the sentence, and $T_{CRF}^{l_{i-1},l_i}$ is the transition matrix storing the score of transferring from $l_{i_1}$ to $l_i$.

The first-order Viterbi algorithm was used to find the highest scored label sequence during decoding. To train the model, the cross-entropy objective function was exploited. Given a manually annotated training data $\{(s_1,y_1),(s_2,y_2),\ldots ,(s_n,y_n)\}$, The loss function is defined as:

11 $$\begin{aligned} L=-\sum _{i=1}^{N}log(P(y_i|s_i))+\lambda \parallel \theta \parallel _2 \end{aligned}$$

where $\lambda$ denotes the $L_2$ regularization parameter and $\theta$ is the all trainable parameters set.

## Experiments

In this section, we conduct a series of experiments to assess the effectiveness of our proposed method.

### Experimental settings

#### Dataset

We use the ancient Chinese NER dataset C-CLUE^29^ to evaluate our proposed model. The C-CLUE dataset originates from all twenty-four history books, which employes a crowdsourcing annotation system. There are 6 types of entities in the initial dataset (https://github.com/jizijing/C-CLUE), but two of them are too few, so this paper excludes them. We use the official divided data set for training, validation and test, and the statistics are shown in Table 1.

**Table 1:** Statistics of the C-CLUE datatset.

Entities	Train set	Validation set	Test set
Person (PER)	11,532	756	859
Location (LOC)	3625	220	236
Office title (OFI)	2252	448	349
Organization (ORG)	2041	40	45
Total	19,450	1464	1489

#### Metric

Following the standard setting, we evaluate the methods using micro-averaged F1 score and also report the precision (P) and recall (R) in percentage. The measurement formula is as follows:

12 $$\begin{aligned} P=\frac{T_{p}}{T_{p}+F_{p}}*100\% \end{aligned}$$

13 $$\begin{aligned} R=\frac{T_{p}}{T_{p}+F_{t}}*100\% \end{aligned}$$

14 $$\begin{aligned} F_1=\frac{2*P*R}{P+R}*100\% \end{aligned}$$

Where $T_p$ represents the number of entities correctly identified by the model, $F_p$ represents the number of entities incorrectly identified by the model, and $F_t$ represents the number of entities that cannot be identified by the model.

All experiments were conducted 5 times with random seeds and the average performance outputs were used for the result reporting and analysis. We conducted all experiments using an Nvidia RTX3090 GPU, Linux operating system (Ubuntu 22.04), and 96GB of system memory for both the all baselines and our proposed method.

#### Hyper-parameters settings

In the training process, the hyper-parameters were tuned on the corresponding development sets. We optimized our model with a stochastic gradient descent (SGD) following Cui and Zhang^31^, the learning rate is set to $2\times 10^{-5}$, and the batch size is 32. The training procedure stopped when the results of the next five validations were not better than the previous best record. Table 2 shows the hyper-parameters used in our experiments.

**Table 2:** Hyper-parameters.

Hyper-parameters	Value	Hyper-parameters	Value
Word emb size (Spanish)	300	Dropout	0.5
Batch size	32	Optimizer	SGD
Momentum	0.9	Learning rate	0.015
Word hidden	400	Learning rate decay	0.05
Gradient clipping	5.0		

#### Baselines

To comprehensively demonstrate our model’s effectiveness, we conduct experiment on two groups of baselines based on whether the model utilizes a dictionary, with all models being state-of-the-art NER models. The first group involves evaluating benchmark models that do not utilize any external information, listed as follows.

- **CNN-CRF**^32^ adopted a standard a convolutional neural network (CNN) encoder on the character sequence, which can obtain its multiple gram features, and used CRF for decoding.
- **BiLSTM-CRF**^33^ used bidirectional LSTMs for encoding and CRF for decoding.
- **TENER**^34^ utilized the Transformer encoder to model the long-range and complicated interactions of sentence.
- **BAC-CRF** used pretarind langugage model **Bert-Ancient-Chinese (BAC)** for encoding.

The second group concentrated on integrating dictionary data into the models, listed as follows.

- **LR-CNN**^35^ incorporated lexicons into CNN-based NER, which leveraged high-level semantics to identify the correct words.
- **Lattice-LSTM**^36^ effectively encoded both individual input characters and possible words using gated recurrent cells, which helps in reducing segmentation errors.
- **NFLAT**^37^ introduced the adapted transformer encoder with a non-flat-lattice structure, which separates lexical fusion from the encoding of contextual features. It can lower the computational demands involved in processing both character–word and word–word interactions during self-attention.

Note that in the experiment, we reproduced all baseline models. To maintain consistency and fairness in comparison, all baselines adopts the same experimental machine environment as the our model. Each experiment was also conducted 5 times with random seeds and the average performance outputs were used for the result reporting and analysis. Furthermore, the static word vectors in the baselines and our model used Tencent Chinese vocabulary embeddings, which offer 200-dimension representations for more than 12 million Chinese words and phrases. These embeddings are derived from extensive pre-training on large-scale corpora.

### Results

Table 3 shows the performances of our method against the baselines. Firstly, it is evident that models which incorporate dictionary information outperform their counterparts that do not. For example, when contrasted with LSTM-CRF, which does not utilize dictionary data, Lattice-LSTM with dictionary integration shows a notable enhancement of nearly $4.25\%$ in F1-score. This highlights the vital importance of dictionary data in bolstering the efficacy of NER models.

Further, in the comparison of models that use pre-trained language model against those that do not, the pre-trained models, namely BAC-CRF and BAC-GNN-CRF, outshine models that lack pre-training, like TENER and NFLAT. This demonstrates that pre-trained language models, which leverage large-scale text datasets, acquire more nuanced contextual information. This enrichment significantly enhances their representation capabilities. The influence of pre-trained language models using various ancient corpora on our model’s performance will be further explored in the “Discussion” section.

Lastly, our proposed model, BAC-GNN-CRF, excels above all others in all metric. Compared to the BAC-CRF model, our model has surpassed it by $3.82\%$ in F1 score. This shows that the two types of external knowledge introduced by our model through GNN, namely dictionary and chapter information, are effective.

**Table 3:** Main results. The results marked with the asterisks are based on our reproduction. A bold number denotes the highest value in that column. The Bold is the best results.

Groups	Methods	P	R	F
Without dictionary	CNN-CRF	56.61	57.12	56.86
	BiLSTM-CRF	56.82	57.2	57.01
	TENER	57.42	59.36	58.37
	BAC-CRF	66.12	76.42	70.89
With dictionary	LR-CNN	60.62	63.65	62.10
	Lattice-LSTM	60.14	62.42	61.26
	NFLAT	62.86	64.09	63.47
Ours	BAC-GNN-CRF	**70.98**	**78.86**	**74.71**

### Discussion

#### Ablation

To investigate the contribution of each component of our model, we conducted ablation experiments on the dataset, and the results are shown in Table 4. The exclusion of the global node (-Global Node), representing chapter information, from the graph structure resulted in a marginal diminution across all evaluative metrics, with precision dropping to $70.57\%$, recall to $78.69\%$, and the F1-score to $74.41\%$. A further decrement in performance was observed upon the elimination of the global node and chapter information from both the encoder and the GNN layer, underscoring the efficacy of integrating chapter embeddings as global nodes within the GNN layer for enhanced utilization of chapter information.

By removing the Graph Neural Network (GNN) layer, there is a more pronounced decline in performance, where precision, recall, and F1-score deteriorated to $67.14\%$, $76.12\%$, and $71.34\%$, respectively. This significant degradation highlights the pivotal role of the GNN layer in the model’s architecture. The -GNN &P excludes both the lexical and the chapter information, referred to as BAC-CRF, which performance drops across all metrics compared to the full model.

The ablation studies have shown that each component of the BAC-GNN-CRF model contributes to its performance. In particular, the GNN layer, emerges as a critical element for precise entity recognition. The global node also aids in maintaining high scores across all metrics, showing its importance in global chapter feature integration. Finally, The best F1-score achieved by the complete model configuration validates the synergistic effect of amalgamating dictionary and chapter information with graph attention mechanisms, delineating a potent strategy for this NER task.

**Table 4:** An ablation study of the proposed model. *–Global node* is the model that remove the global node in the graph. *–Global node &P* refers to remove the chapter information, only incorporating the dictionary knowledge. *–GNN* refers to remove the GNN layer. *–GNN &P* refers to remove the GNN and chapter, namely BAC-CRF.

Methods	P	R	F
BAC-GNN-NER	70.98	78.86	74.71
–Global Node	70.57	78.69	74.41
–Global Node &P	70.35	78.45	74.18
–GNN	67.14	76.12	71.34
–GNN &P	66.12	76.42	70.89

#### Impact of different pre-trained language model

Table 3 has illustrated the significant role of pre-trained language models in enhancing the performance of NER in Ancient Chinese Books. Researchers have proposed multiple ancient Chinese pre-trained language models such as Roberta-Classical-Chinese (Roberta-Classical-Chinese: https://huggingface.co/KoichiYasuoka/roberta-classical-chinese-base-char), GuwenBert-Base (GuwenBERT https://github.com/ethan-yt/guwenbert), SikuBert (SikuBERT https://huggingface.co/SIKU-BERT/sikubert) and Bert-Ancient-Chinese (Bert-Ancient-Chinese https://github.com/Jihuai-wpy/bert-ancient-chinese). Since these pre-training models use different ancient text training corpora, they have different impacts on the performance of our proposed model. Table 5 presents the performances of different pre-trained language model over our framework. While Bert-Base-Chinese (Bert-Base-Chinese https://huggingface.co/bert-base-chinese) was developed with modern text, other models were honed on various classical texts. We can see that language models pre-trained on classical Chinese greatly enhance the model’s performance compared to Bert-Base, checking that pre-trained language models on classical Chinese can enhance the representation of ancient texts. Furthermore, of the models pre-trained on classical texts, Bert-Ancient-Chinese(BAC), used by our our model, achieves the best performance. This could be attributed to the inclusion of the experimental dataset within the pre-training corpora.

**Table 5:** The performances of different pre-trained language models.

Methods	P	R	F
Bert-Base-Chinese	60.45	65.15	62.71
Roberta-Classical-Chinese	64.12	72.41	68.01
GuwenBert-Base	67.37	74.28	70.66
SikuBert	69.93	76.52	73.08
Bert-Ancient-Chinese	70.98	78.86	74.71

#### Comparison on entity categories

**Figure 2:**
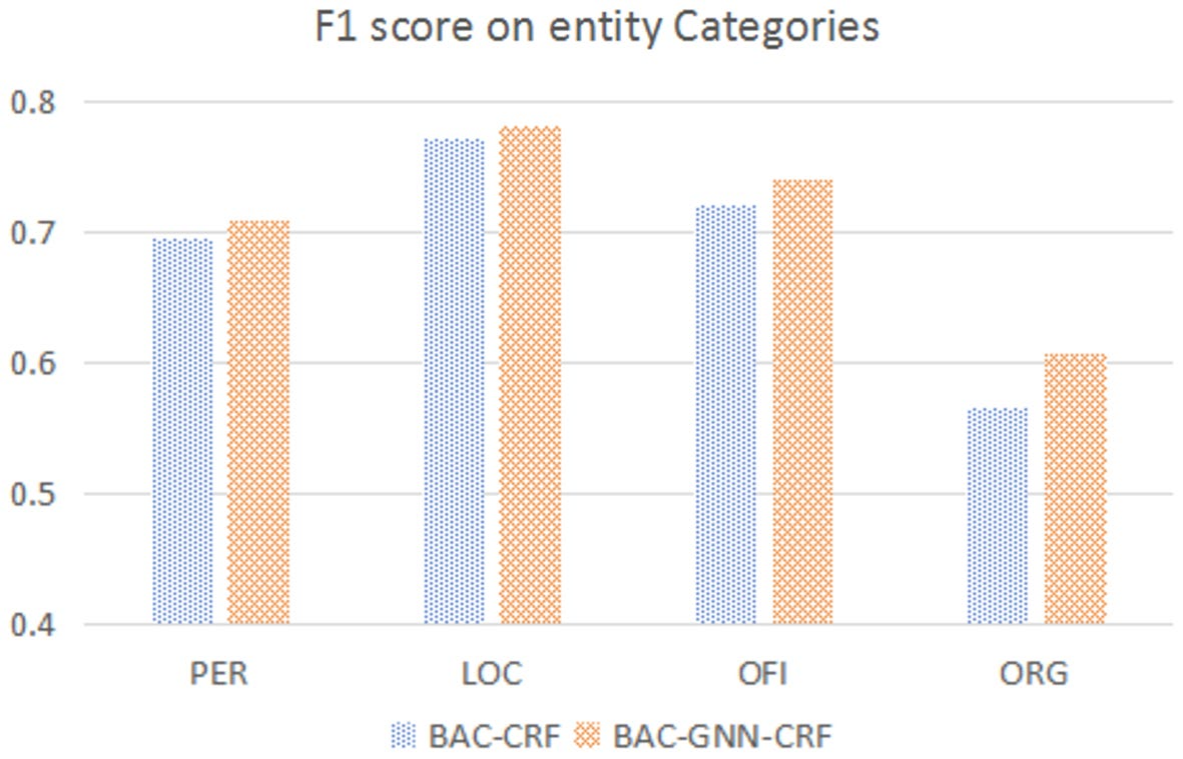
Performance comparison across various entity categories between BAC-CRF and BAC-GNN-CRF.

The Fig. 2 shows a comparative analysis of the F1 score between our model and the baseline BAC-CRF, across four different entity categories: PER (Person), LOC (Location), OFI (Office title), and ORG (Organization). We can see that our model outperforms the BAC-CRF in every category. This suggests that the integration of GAT in the BAC-CRF framework helps to better capture the context and semantic of texts, which is beneficial for the task of named entity recognition. The biggest improvement can be seen in the ORG category, with an increase of 4.21% in the F1-score. This could indicate that the BAC-GNN-CRF model is particularly better at handling the complexities associated with organizational names, which may be due to more effective disambiguation of entities facilitated by the integration of an external dictionary.

#### Performance against entity length

**Figure 3:**
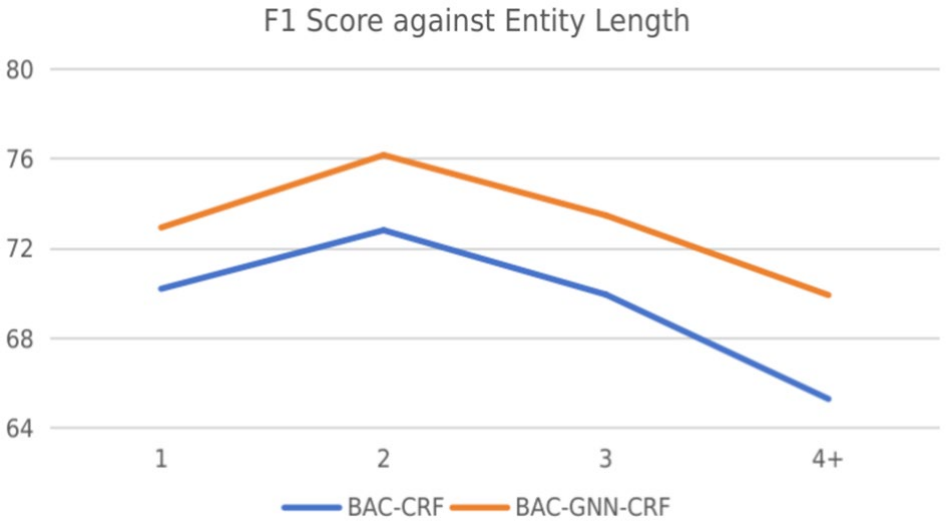
Performance comparison against entity length between BAC-CRF and BAC-GNN-CRF.

We also analysis the NER performance relative to the length of entities. We categorize the entity lengths into four categories: 1, 2, 3 and 4+ (i.e., $\ge 4$). Figure 3 shows the results, where the F1-scores of both the BAC-CRF and our model are offered. For entities composed of a single character, both models exhibit diminished performance in contrast to entities spanning two characters. This observation suggests that entities of a single character in ancient Chinese texts are likely to present a higher degree of ambiguity.

A significant enhancement in F1-score is evident for both models when processing bi-character entities, underscoring the likelihood that entities of this length offer more distinct contextual cues, thereby facilitating more accurate identification by the models and mitigating ambiguity. However, as entity length extends beyond two characters, a decline in performance is observed for both models. This trend highlights the increasing challenges associated with delineating the boundaries of more extended entities.

Our model consistently outperforms the BAC-CRF across all evaluated conditions, illustrating its capability in enhancing the representation of ancient Chinese text. This advantage is attributed to the strategic incorporation of external dictionaries and the chapter-level context.

**Table 6:** Four samples outputted by the BAC-CRF and the BAC-GNN-CRF. The case in Gold row is composed of annotated sentence with its corresponding chapter title. The outputs of both models are the predicted sentences without the chapter information.

Case 1	
Gold	广顺元年，实录成，[纬]_PER求迁官不得，由是怨望。＃ 新五代史>卷五十七-杂传第四十五>贾纬 （ In the first year of Guangshun, Wei completed his official service record, but his request for a transfer to a different official position was not granted, leading to feelings of resentment and disappointment.＃ New History of the Five Dynasties>Volume 57-Miscellaneous Biography 45>Jia Wei )
BAC-CRF	广顺元年，实录成，[纬]_PER求迁官不得，由是怨望。
BAC-GNN-CRF	广顺元年，实录成，[纬]_PER求迁官不得，由是怨望。
Case 2	
Gold	[翰林学士]_OFI[徐台符]_PER以为不可，数以非[纬]_PER。＃ 新五代史>卷五十七-杂传第四十五> 贾纬 ( Xu Taifu, an academician of the Hanlin Academy, believed it was inappropriate and repeatedly pointed out Wei’s mistakes.＃ New History of the Five Dynasties>Volume 57-Miscellaneous Biography 45>Jia Wei )
BAC-CRF	[翰林学士]_OFI[徐台符]_PER以为不可，数以非[纬]。
BAC-GNN-CRF	[翰林学士]_OFI[徐台符]_PER以为不可，数以非[纬]_PER。
Case 3	
Gold	[松]_PER举[进士]_OFI，[后唐]_ORG时，历[刑部郎中]_OFI，[唐]_ORG末，从事方镇。＃ 新五代史>卷五十七-杂传第四十五>王松 （Song passed the imperial examinations and became a Jinshi. During the Later Tang Dynasty, he once served as a Director in the Ministry of Punishment. By the end of the Tang Dynasty, he held the position of a military commander, guarding a region. ＃ New History of the Five Dynasties>Volume 57-Miscellaneous Biography 45>Wang Song ）
BAC-CRF	[松]_PER举[进士]_OFI，后[唐]_ORG时，历[刑部]_ORG[郎中]_OFI，[唐]_ORG末，从事[方镇]_LOC。
BAC-GNN-CRF	[松]_PER举[进士]_OFI，[后唐]_ORG时，历[刑部郎中]_OFI，[唐]_ORG末，从事[方镇]_LOC。
Case 4	
Gold	命[中书郎]_OFI[李]_PER 于树下造诏。＃ 北史>卷五十六-列传第四十四>魏收 （Ordered Li Yin, the secretary of the imperial court, to draft the imperial edict under the tree. ＃ Northern History>Volume 56- Biography 44>Wei Shou）
BAC-CRF	命[中书郎]_OFI [李音]_PER 于树下造诏。
BAC-GNN-CRF	命[中书郎]_OFI[李音]_PER于树下造诏。

#### Case study

We conduct a case study. Table 6 shows four cases in the test sets. we can see that the “纬 (Wei)” is correctly recognised by the BAC-CRF and the BAC-GNN-CRF models in case 1, while the BAC-CRF makes a mistake and our model still does it correctly in case 2. The reason is that the two sentences originate from the same chapter:“新五代史> 卷五十七-杂传第四十五> 贾纬. （New History of the Five Dynasties>Volume 57-Miscellaneous Biography 45> Jia Wei)” ,which is integrated into our model as a global node and can help alleviate the ambiguity of word “纬(Wei)”.

**Figure 4:**
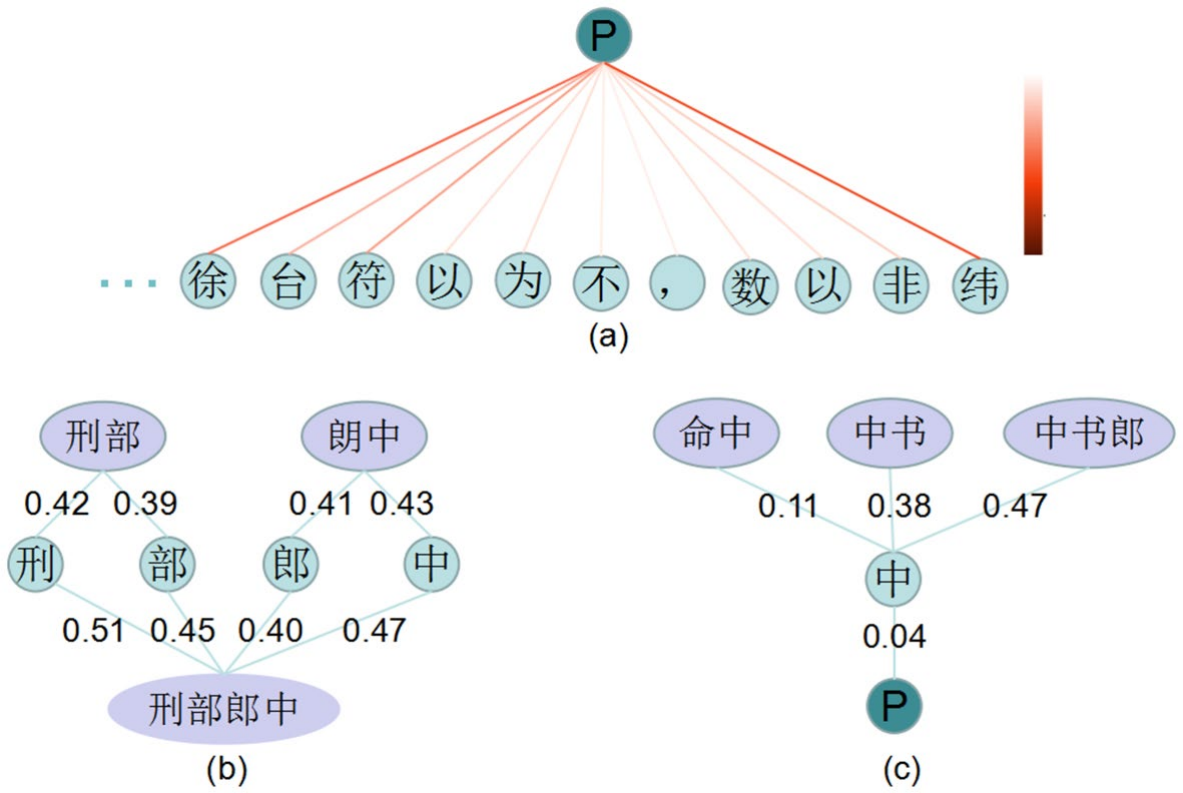
The visual analyses of graph attention. (**a**) The attention coefficients related to the global node on case 2. The darker the color, the greater the weight value. (**b**) The attention coefficients for the entity “刑部郎中” on case 3. (**c**) The impact of noised word on case 4. * All values are rounded to two decimal places.

In case 3, the BAC-CRF erroneously split “刑部郎中(Director in the Ministry of Punishment)” into two separate entities: “刑部(the Ministry of Punishment)” and “郎中(doctor)” . However, as “刑部郎中” is found in the external dictionary as a lexical item, our model was able to identify it correctly. This illustrates the efficacy of external dictionaries in enhancing the accurate delineation of entity boundaries. On the contrary, since the term “镇(Town)” frequently appears in geographic names, both models erroneously classified ’方镇(Fang Town)’ as a location entity. This misclassification requires more contextual information and external knowledge to be solved. Case 4 provides an example of a noise word and the associated analysis is shown in Fig. 4c.

Furthermore, we present three visual analyses of the graph attention module in Fig. 4. Figure 4a displays the attention coefficients related to the global node in case 2, showing that characters within a specific entity are assigned higher weights, while other commonly occurring characters receive lower weights. Figure 4b shows the attention weights for the entity “刑部郎中” in case 3, demonstrating that the semantics of the matching words are effectively distributed across the individual characters “刑”, “部”, “郎”, and “中”. This enables our model to accurately identify the entity “刑部郎中”. Figure 4c demonstrates the effect of a noisy word in case 4. The noisy word “命中”, which could potentially disrupt entity recognition, is assigned a low weight, enabling our model to correctly identify the entity due to the attention module.

## Conclusion

In this paper, we introduce a semantic enhanced graph neural network model, namely BAC-GNN-CRF, specifically tailored for NER in ancient Chinese books. Our approach employs a graph-based framework to integrate two distinct forms of external knowledge: dictionary-level and chapter-level information. This integration is aimed at augmenting the semantic representation of the texts and mitigating ambiguity. Additionally, we leverage Graph Attention Networks (GAT) to diminish the influence of matching noise and to more effectively incorporate the aforementioned external knowledge. The experimental evaluations conducted on the C_CLUE dataset substantiate the effectiveness of our model, evidencing substantial improvements and achieving the best score compared to several state-of-the-art dictionary-augmented models. In the future, we intend to broaden the scope of our model by integrating additional forms of external knowledge and by exploring more advanced machine learning architectures to further enhance NER capabilities within the domain of ancient Chinese literature. All codes and resources are released at the website: https://github.com/qtxcm/BAC-GNN-CRF.

## Data Availability

The datasets that support the findings of this study are openly available in the C_CLUE database (https://github.com/jizijing/C-CLUE).
